# Rise of inflation and formation of interest rate on loans in industrial sector: A VECM approach to assess the impact on total industrial production from evidence of Bangladesh

**DOI:** 10.1016/j.heliyon.2024.e24976

**Published:** 2024-01-24

**Authors:** Adnan Chowdhury, Tamanna Siddiqua Ratna, Tanzin Akhter, Syed Far Abid Hossain

**Affiliations:** a Bangladesh Bank, Motijheel C/A, Dhaka, 1000, Bangladesh; b Department of Quantitative Sciences, International University of Business Agriculture and Technology (IUBAT), 4 Embankment Drive Road, Sector-10, Uttara, Dhaka, 1230, Bangladesh; c BRAC Business School, BRAC University, 66 Mohakhali, Dhaka, 1212, Bangladesh

**Keywords:** Weighted average interest rate on advance (WAIRA), Inflation, Industrial production index, Industrial development, Johansen test, VECM, Bangladesh

## Abstract

Industrial development is the prerequisite for sustainable economic growth. This study has examined the impact of the interest rate imposed on advances in the small and medium enterprise (SME) industrial sector, the large industrial (LI) sector, and inflation on the total industrial development of Bangladesh. For this purpose, we have used monthly data from January 2015 to June 2021. The weighted average interest rate of advances (WAIRA) is used as a substitute for the interest rate imposed on advances that were sourced from Bangladesh Bank (BB). We have applied Johansen co integration and the VECM technique to investigate long- and short run relationships, and the results have revealed the existence of both. We have observed that, in the short run, only WAIRA on SME industries has a significant negative impact on total industrial development. In the long run, inflation and WAIRA on SME and large industry have a significant impact on industrial development. The long run relationships have indicated that, inflation and WAIRA in the SME sector have a negative influence on total industrial development, but WAIRA in the large industry (LI) sector has a positive influence. Finally, considering the entire situation of the economy of Bangladesh, especially the growing industrial sector, these findings are highly momentous for policy implications and achieving sustainable development in this sector.

## 1 Introduction

Bangladesh has gone through significant structural changes in its economy during the last 50 years. When it was founded in 1971, Bangladesh was described as an agrarian-based country. At that time, agriculture's contribution to the total economy was more than 50 %. According to World Bank statistics, the contribution of the agricultural sector to the economy of Bangladesh was 51.03 %, whereas the contribution of the industrial sector was only 7.68 %. During the last five decades, both the agricultural and industrial sectors have gone through indicatory transformations, but if we look at the present situation, rapid growth in the industrial sector has been observed. The establishment of industrial parks, an increase in foreign direct investment, and rapid growth in small and medium enterprises have all been indicators of the improvement of the industrial sector.

The industrialization of Bangladesh started with the development of the ready-made garment sector, but later, in the early 1990s, many sectors began their voyage, effectively contributing to Bangladesh's economic progress. A significant component of the investment industry is reliant on foreign direct investment. Foreign investment has been a major driver of industrial expansion in Bangladesh. In Bangladesh, local investors are also a major source of revenue and entrepreneurship. They have a comprehensive awareness of the local market, which enables them to recognize opportunities and overcome obstacles.

Two of the most important variables in the expansion of the industrial sector are the facilitation and implementation of a proper industrial environment. Given the current global competitive environment, both the government and the private sector are playing an increasingly crucial role in the country's industrialization. As a result, the government's Ministry of Industries has taken on the role of a facilitator. Aside from that, the government has implemented a number of positive and timely business reforms as well as liberalized trade, allowing private entrepreneurs to profitably and freely develop and operate industrial firms, which are reflected in the present industrial statistics of Bangladesh. But for local investors, one of the most important sources of financial support for expanding industries is the lending of money from government and non-government banks and financial institutions. The available interest rate for this type of loan is a critical metric for gaining access to economic and financial development.

Short-term investment decisions in developing countries might be affected by interest rate increases. Higher interest rates make borrowing more expensive, reducing investment, whereas lower interest rates make borrowing cheaper, encouraging investment [1]. Keynesian macroeconomic theory also predicts an inverse link between interest rates and investment. Interest rates are a major determinant of industrial development [2] as well as financial growth. Recent empirical studies have examined the relationship between interest rates, public and private investment, and financial or economic development. The loan interest rate is affected by GDP growth and manufacturing [3]. Nguyen and Trinh [4] used the ARDL framework to analyze the dynamic impact of public and private investment on economic growth. Their research demonstrates that public investment strongly affects economic growth. Jacobe et al. [5] estimated a panel VAR model that confirmed real interest rates had a shock on economic growth. Short-term interest rate effects on economic growth have also been discovered [6]. Hatmanu et al. [6] used autoregressive distributed lag methods to investigate how Euro zone interest rates affect Romanian economic growth. These findings suggest that short-term economic growth is negatively impacted by interest rates. George- Anokwuru and Bosco [7] examined the effect of interest rates on the industrial sector. They used the ARDL Bound test, which confirmed the positive relationship between interest rates and industrial output. They have also considered inflation, which is negatively related to industrial output. The interest rate has had a substantial impact on the expansion of the economy over the long run [8]. Hossin [9] has analyzed the effect of interest rate reforms on the financial development of Bangladesh. That study confirmed that interest rates and financial development have bi-directional causality using Granger causality. Mohsen et al. [10] conducted a study to determine how interest rates affect the economic growth of Bangladesh. According to the author, interest rates drive economic growth.

Bangladesh's industrial sector can be divided into two major categories: large industries and small and medium-sized industries (SMEs). The large industry sector consists mainly of two parts: manufacturing and service industries. The manufacturing industries of Bangladesh consist of mainly tobacco processing industries, rubber and plastic industries, leather product manufacturing industries, textile mills, pharmaceutical industries, chemical and chemical products industries, cement factories, ceramic industries, brick manufacturers, sand elevators, glass and glassware product factories, steel engineering and metallic products industries, and assembling industries, whereas the service industries consist of mainly transport, publishing, telecommunication, entertainment, health services, IT-based activities, tourism, etc., according to the Bangladesh Bank's scheduled bank statistics.

The government is consistently taking comprehensive measures to develop and prosper in all industrial sectors of the country. The administration unveiled the “National Industrial Policy—2016″ in order to speed up the country's industrialization. The government is continuing its efforts to attain this goal by partnering with banks and other financial institutions to provide loans and other auxiliary support. As a result, the amount of industrial loan distribution and recovery is increasing. In July–September 2021, credit flow to the industrial sector increased by more than 12 % year on year, indicating that economic activity has recovered with the pandemic scenario turning the corner. According to Bangladesh Bank data, bank loans to the industry were disbursed at a rate of Tk 1,06,596 crore in 2021, compared to Tk 94,849 crore in the same period in 2020. However, the quarter's distribution fell by Tk 644 crore from Tk 1,07,241 crore in the April–June quarter of 2021. Due to the pandemic, bank loan disbursements to the industrial sector were reduced in June 2020. The situation has improved as a result of the government's various stimulus loans. However, overall lending to the private sector increased.

Cottage, Micro, Small, and Medium Enterprises (CMSMEs) are seen as a promising area of industrial growth. By fostering and developing corporate activity, this sector has played a vital role in attaining economic growth and earning foreign funds. Banks and non-bank financial institutions (NBFIs) have stepped forward to help finance and develop the SME sector, which is being closely monitored and supervised by the Bangladesh Bank. At the end of September 2020, the total net outstanding loans and advances in the SME sector were Tk. 1,90,969.83 crore (BB). Bangladesh Bank currently operates five SME refinance schemes with the help of the government and various development partners, including the refinance scheme for agro product-processing industries in rural areas, the Bangladesh Bank Fund, the Bangladesh Bank Women Entrepreneurs Fund, the New Entrepreneurs Fund, and the Islamic Shariah-Based Fund.

The novelty of this study lies in the fact that we examine the influence of separately imposed interest rates on SME and the LI on industrial development, whereas the majority of scholars analyze the impact of interest rates on economic or financial growth. In general, the interest rates on SME loans and LI loans are affected by the overall level of interest in the economy, which is regulated by the central bank's monetary policy. Credit, such as SME loans and LI loans, is an essential source of finance for firms and can influence their capacity to invest and expand. Credit availability can have a substantial effect on industrial production [11]. Higher interest rates also discourage high-risk investments [12]. Consequently, an increase in interest rates affects both investment and consumer spending. As an emerging country, many researchers have already focused their research on the challenges facing the SME industry in Bangladesh and suggested valuable policies. The effect of increasing SME loan rates has a significant impact on economic growth as a whole. SME financing is vital not only for launching a SME business, but also for fostering its growth [13]. This study also asserts that despite their great potentials, SMEs cannot enter the manufacturing business due to finance issues. And one of the major impediments to a growing SME industry is getting funding at high interest rates. Access to capital is the greatest barrier to the development of small and medium-sized enterprises in both developing and industrialized nations ([[14], [15], [16]]). The growth of industrial production in emerging developing nations such as Bangladesh relies heavily on the SME sector. According to Qamruzzaman and Jianguo [17] SME financing innovation is positively related to both long-run and short-run growth in Bangladesh. Ali et al. [18] had pointed out a gap between the demand and supply of SME lending. They found that in addition to the interest on institutional credit, SMEs have to endure some other expenses besides the interest on institutional credit.

Inflation is an important macroeconomic variable. The inflation rate is a significant factor in the development and expansion of the industrial sector of a country. The rate of inflation is a crucial metric that has significant implications for the economy. High rates of inflation, in particular, often deter investment and result in lower long-term growth. A very low inflation rate also indicates the availability of fewer goods and services than required and shows slow economic growth. Inflation can affect industrial production by raising input costs and decreasing output value. Stable and low inflation boosts industrial production and economic growth [11]. The monetary policy, where interest rates and other instruments are used by the central authorities to influence the economy, can affect the interest rate, inflation, and industrial production [19].

Several empirical studies reveal the effects of inflation in different ways. GDP buffers financial development from inflation [20]. According to Ehigiamusoe et al. [21], the inflation rate promotes economic growth. Despite the fact that lower inflation has accelerated economic growth [22]. Again, the behavior of inflation in relation to economic or financial growth may differ depending on long-term or short-term considerations. Svigir and Milos [23] discovered a long-run negative association between inflation and financial growth that coexists with a short-run positive relationship. Kim and Lin [24] have investigated the factors that influence inflation and economic growth in Pakistan. According to their findings, inflation has a detrimental impact on economic growth in the short run. However, scholars emphasize financial development or economic growth, although industrial development might be regarded an essential component in determining the state of the economy. Industrial growth optimizes price reduction, job creation, national income, technology, transportation, agriculture, production, trade, and all other economic activities. In addition, it enhances employment possibilities, training, educational growth, labor productivity, regional development, resource allocation, and usage [25]. However, there is no explicit econometric study for Bangladesh that can infer the impact of interest rates and inflation on industrial growth as a whole. This gap in the literature motivated us to conduct the present investigation.

There are few studies that have focused on industrial development while taking into account various macroeconomic control variables. Maroof et al. [26] defined the pro-poor group in their study and established the association with inflation. The output of their study has suggested that a low inflation rate has a significant association with pro-poor growth, whereas the term “pro-poor” is defined as growth that benefits the poor proportionately more than the non-poor. Son and Kakwani [27], utilizing an ARDL-bounding testing strategy to analyze the short-run and long-run causal link between inflation, investment, and growth in Tanzania, discovered a unidirectional causal flow from inflation to development. Odhiambo [28] conducted a study to determine the effect of monetary inflation on the investment decisions of Jordanian industrial firms. Azra et al. [25] examined the industrial growth of south Asian nations, including Bangladesh. They used Panel ARDL to explore the long-term relationship. There was evidence for Bangladesh that, in the short term, inflation has a strong positive effect on industrial production, as measured by industrial value added as a proxy variable. According to the findings of the long-term estimations, inflation has a positive and substantial impact on the industrial growth of South Asian economies [28]. Shimu and Islam [29] have evaluated the impact of inflation on the expansion of Bangladesh's RMG industry. As a result, the impact of our concerned macroeconomic variables on Bangladesh's industrial development is superficial; thus, this study aims to fill this knowledge gap. This study examines how inflation and interest rates (imposed on SME and LI) affect overall industrial production in Bangladesh to explain industrial growth. Therefore, the following questions are addressed in this research:

1. How do imposed interest rates in SME and LI affect industrial development in the short run?
2. How does inflation affect industrial development in the short run?
3. Do inflation and interest rates affect industrial development in the long run?
4. If there is a long-run association, how do these dynamic relationships can be explained?

The rest of this study is divided into five sections to answer these stated questions. Section 2 describes the data and methodology, Section 3 concentrates on econometric model specification, Section 4 explains empirical results and findings and focuses on robustness checking, Section 5 concludes the result and suggests some policies, and Part 6 shows the limitations of this study.

## 2 Data and methodology

Monthly data from January 2015 to June 2021 were extracted for this study. We have used the following variables: the industrial production index as a proxy variable for total industrial development (TIPI), the interest rate imposed on advances in the small and medium enterprise industry sector (SME), the interest rate imposed on the large industry sector (LI), and inflation. The weighted average interest rate on advances (WAIRA) is used as a substitute for the interest rate imposed on advances. All variables were collected from the Bangladesh Bank and transformed into natural logarithms.

Since the aim of this research is to analyze the nexus between inflation, levying interest rates on industrial sectors, and industrial development, the following econometric model is specified:

(1) $$TIPI_{t}=\alpha_{0}+\alpha_{1}LIWAIRA_{t}+\alpha_{2}SMEWAIRA_{t}+\alpha_{3}INF_{t}+\varepsilon_{t}$$

All variables are considered in their logarithmic form to remove stochastic error and determine the elasticity of study variables. Therefore, the following equation (2) is the natural logarithm of equation (1):

(2) $$LnTIPI_{t}=\alpha_{0}+\alpha_{1}LnLIWAIRA_{t}+\alpha_{2}LnSMEWAIRA_{t}+\alpha_{3}LnINF_{t}+\varepsilon_{t}$$

Where, subscript t denotes the year, $\varepsilon_{t}$ indicates the error term, LnTIPI is the natural logarithm of total industrial production index, LnLIWAIRA and LnSMEWAIRA are natural logarithm of weighted average interest rate of advances on large industry sector and small and medium enterprise industry sector respectively, LnINF is the natural logarithm of inflation rate.

This empirical study requires some pre-test to estimate equation (2). As a result, we checked the econometric validation of our proposed model by using the Breusch – Pagan [29] test to check the heteroscedasticity and the Shaprio-Wilk test [30] to check the normality respectively.

## 3 Econometric model specification

To specify the econometric model, in this research, the unit root test is led by adopting the Augmented Dickey Fuller (ADF) test [31] to identify the non-stationarity features of the study variables. Unit root checking is one of the vital parts of econometric analysis, as non-stationary time series lead to spurious regression. The null hypothesis of an ADF test is that the data contain a unit root. When the series becomes stationary, it must determine whether or not the variables are integrated, as well as the integrated level. This study determines the best lag by using various lag selection criteria.

The co-integration analysis has been conducted, which helps trace the long-run relationship among variables. This study used the Johansen co-integration test [32] to ascertain the long-run relationship between variables. According to the Johansen co-integration test, the Vector Error Correction Model (VECM) is applicable if the co-integration test can establish the presence of long run dynamics between variables. VECM is used to assess the long-term and short-term associations between variables. The details of the model specification are given below.

### 3.1 Augmented Dickey Fuller (ADF) test

To evaluate if the time series data has a unit root, an augmented Dickey–Fuller (ADF) test was utilized. Equation (3) represents the theoretical model [31],

(3) $$\Delta Y_{t}=\beta_{0}+\beta_{1}t+\delta Y_{t-1}+\sum_{i=1}^{p}\alpha_{i}\Delta Y_{t-i}+\varepsilon_{t}$$

where, Δ is a first difference operator, $Y_{t}$ denotes any time series variables (interest rate imposed on advances in the SMEs, the interest rate imposed on the large industry sector (LI), inflation), $\beta_{0}$ is a constant, $\beta_{1}$ is a coefficient on a time trend, p isthe lag order of the autoregressive process, and $\varepsilon_{t}$ denotes the random error term. The tested hypotheses of the above model (Equation (3)) are,

$$H_{0\;}:\delta =0\;or\;Y_{t}\;is\;non-stationary\text{.}$$

$$H_{1}:\delta \neq 0\;or\;Y_{t}\;is\;Stationary\text{.}$$

The suggested test statistic for this test is $\tau \left(\text{ADF}\right)=\frac{\hat{\delta }}{\text{SE}(\hat{\delta )}}$ which follows the t-distribution and the decision of this test is determined in current study by considering 5 % level of significance.

### 3.2 Johansen's co-integration test

By creating spurious regression, trended time series can cause major problems in empirical econometric studies. Johansen's co-integration test has been used to determine the presence of co-integrating vectors. The following model, described by equation (4), is used to carry out this test [32],

(4) $$Y_{t}=\alpha +\omega_{1}Y_{t-1}+\cdots \cdots \cdots +\omega_{p}Y_{p-1}+\vartheta_{t}$$

where, $Y_{t}$ is an (n x 1)-dimension vector of variables ∼I(1), and $\vartheta_{t}$ is an (n x 1)-dimension vector of innovations.

Therefore, equation (4) can be re-written using as equations (5), (6), (7).

(5) $$\Delta Y_{t}=\alpha +\Pi Y_{t-1}+\sum_{i=1}^{p-1}\Gamma_{i}\Delta Y_{t-i}+\vartheta_{t}$$

where,

(6) $$\Pi =\sum_{i=1}^{p}\omega_{i}-I$$

(7) $$\Gamma_{i}=-\sum_{j=i+1}^{p}\omega_{j}$$

If Π has the rank r<n, then there will be (n x r) matrices γ and δ, both having rank r in a way that Π = $\gamma \delta^{\prime }$, whereas $\delta^{\prime }Y_{t}$ is stationary. Two likelihood ratio tests (LRTs) utilized to test the significance of the reduced rank of Π are defined as below.

#### 3.2.1 Trace test

The likelihood ratio test about the trace matrix has considered the test statistic, which is defined in the following equation (8) [32],

(8) $${\hat{\lambda }}_{trace}=-T\sum_{i=r+1}^{n}\ln\left(1-{\hat{\lambda }}_{i}\right)$$

where, T isthe sample size and ${\hat{\lambda }}_{i}$ denotes estimated eigen values ranked from largest to smallest. The hypothesis of this test can be defined as,

$$H_{0\;}:There\;are\;at\;most\;r\;co-intregating\;equations\;\text{.}$$

$$H_{1}:there\;are\;n\;co-intregating\;equations$$

#### 3.2.2 Maximum eigen value test

The likelihood ration test about the Eigen value matrix has considered the following test statistic which is defined by following equation (9) [32].

(9) $$J_{\max}=-T\;\ln\left(1-{\hat{J}}_{r+1}\right)$$

where, N and ${\hat{J}}_{i}$ are defined the same as for the trace test.

### 3.3 VECM model

If either Eigen value test or trace test results co-integrating equation then there exists a long-run association. Let, two I(1) series x and y are co-integrated, then there are unique α_0_ and α_1_ and presented by the following equation (10),

(10) $$U_{t}=Y_{t}-\alpha_{0}-\alpha_{1}X_{t}$$

Here, equation (10) is I(0). If y is the dependent variable and x is an exogenous regressor in the single-equation model of co-integration, the error-correction model is stated in equations (11), (12),

(11) $$△y_{t}=\beta_{0}+\beta_{1}△x_{t}+\lambda u_{t-1}+{Ԑ}_{t}$$

(12) $$=\beta_{0}+\beta_{1}△x_{t}+\lambda \left(y_{t}\;–\;\alpha_{0}-\alpha_{1}x_{t}\right)+{Ԑ}_{t}$$

But in the specification of a vector auto regressive model, all variables are regarded identically, therefore, for two variables with lag 1 and one co-integrated equation system is presented in equations (13), (14) as,

(13) $$△y_{t}=\beta_{y0}+\beta_{yy1}△y_{t-1\;}+\beta_{yx1}\;△x_{t-1}+\lambda_{y}\left(y_{t-1}\;–\;\alpha_{0}-\alpha_{1}x_{t-1}\right)+{Ԑ}_{t}^{y}$$

(14) $$△x_{t}=\beta_{x0}+\beta_{xy1}△y_{t-1\;}+\beta_{xx1}\;△x_{t-1}+\lambda_{x}\left(y_{t-1}\;–\;\alpha_{0}-\alpha_{1}x_{t-1}\right)+{Ԑ}_{t}^{x}$$

The error-correction coefficients, λ measure each variable's response to the previous period's degree of divergence from long-run equilibrium, and we expected it will be −2 < λ < 0 [33].

For our current study, considering VECM framework short run equations are expressed below:

(15) $$\Delta LnTIPI_{t}=\beta_{1}+\sum_{i=1}^{k}\lambda_{1i}\Delta LnLIWAIRA_{t-1}+\sum_{i=1}^{k}\gamma_{1i}\Delta LnSMEWAIRA_{t-1}+\sum_{i=1}^{k}\mu_{1i}\Delta LnINF_{t-1}+\sum_{i=1}^{k}\pi_{1i}\Delta LnTIPI_{t-1}+\delta_{1}\varepsilon_{t-1}+e_{1t}$$

(16) $$\Delta LnLIWAIRA_{t}=\beta_{2}+\sum_{i=1}^{k}\lambda_{2i}\Delta LnLIWAIRA_{t-1}+\sum_{i=1}^{k}\gamma_{2i}\Delta LnSMEWAIRA_{t-1}+\sum_{i=1}^{k}\mu_{2i}\Delta LnINF_{t-1}+\sum_{i=1}^{k}\pi_{2i}\Delta LnTIPI_{t-1}+\delta_{2}\varepsilon_{t-1}+e_{2t}$$

(17) $$\Delta LnSMEWAIRA_{t}=\beta_{3}+\sum_{i=1}^{k}\lambda_{3i}\Delta LnLIWAIRA_{t-1}+\sum_{i=1}^{k}\gamma_{3i}\Delta LnSMEWAIRA_{t-1}+\sum_{i=1}^{k}\mu_{3i}\Delta LnINF_{t-1}+\sum_{i=1}^{k}\pi_{3i}\Delta LnTIPI_{t-1}+\delta_{3}\varepsilon_{t-1}+e_{3t}$$

(18) $$\Delta LnINF_{t}=\beta_{4}+\sum_{i=1}^{k}\lambda_{4i}\Delta LnLIWAIRA_{t-1}+\sum_{i=1}^{k}\gamma_{4i}\Delta LnSMEWAIRA_{t-1}+\sum_{i=1}^{k}\mu_{4i}\Delta LnINF_{t-1}+\sum_{i=1}^{k}\pi_{4i}\Delta LnTIPI_{t-1}+\delta_{4}\varepsilon_{t-1}+e_{4t}$$

From the above equations (15), (16), (17), (18), Δ denotes the first difference and λ,γ,μ,π are the coefficients of the lagged predictor variables and $\varepsilon_{t-1}$ indicates the error term. For this research work we consider total industrial production as the dependent variable, so our concern is only equation (3). Furthermore, various diagnostic tests were conducted to check the reliability of the dynamic model. We applied Jarque-Bera test [34] to check the error normality, and LM test [35] to check the presence of autocorrelation.

### 3.4 Fully Modified Ordinary Least Squares method (FMOLS)

The present research utilizes the Fully Modified Ordinary Least Squares (FMOLS) method to assess the robustness of long-run parameters derived from the VECM model. The FMOLS method was developed by Phillips and Hansen [36]. In order to estimate the long-run parameters, FMOLS utilizes a semi-parametric framework ([[37], [38], [39]]). The FMOLS method provides consistent coefficients for small sample sizes, evaluates the reliability of the findings, and addresses the endogeneity problem, serial correlation factors, and long-run coefficients of heterogeneity ([39,40]). To apply the FMOLS methods, all study variables should be integrated in order one, I(1) [41].

## 4 Empirical results and findings

The findings of our current study are basically divided into two portions. In the first portion, econometric validation tests are applied to confirm data validation, and then to quantify the dynamic effect of study variables, the rest of the analysis has been done under the Vector Error Correction Model (VECM) framework. In the second portion, we have performed model validation and robustness tests for our selected dynamic model.

### 4.1 Econometric validation & VECM framework findings

The results of the data validation tests are shown in Table 1 and the findings confirm that residuals are normally distributed along with constant variance.

**Table 1:** Diagnostic tests result.

Test	p-value	Null Hypothesis (H_0_)	Decision
Shapiro-Wilk	0.22	Residuals are normally distributed.	H_0_ is accepted
Breusch-Pagan	0.06	Residual variances are constant	H_0_ is accepted

To verify the stationary characteristics of our study variables, we have used the ADF test. Findings of the ADF test are summarized in Table 2. The null hypothesis of the ADF test is that there is a unit root in the data, whereas the alternative hypothesis describes the data as stationary. The result of the ADF test has suggested that the variables are integrated at order one, I(1), at the 5 % level of significance. These supports applying the Johansen co-integration test to confirm the long-run association among variables. Hence, Table 3 points out the optimal lag for our study, and the results suggest lag 2 for further analysis.

**Table 2:** Unit Root test results.

Variables	ADF				Order of Integration I(0) or I(1)
	Level		1st Difference		
	t-statistic	*P*-value	t-statistic	*P*-value	
LnTIPI	−1.683	0.4357	−12.067	0.0001**	I(1)
LnLIWAIRA	−0.561	0.8795	−7.556	0.0000**	I(1)
LnSMEWAIRA	−0.287	0.9272	−8.254	0.0000**	I(1)
LnINF	−2.429	0.1372	−3.385	0.0145**	I(1)

Note: ADF refers the Augmented Dicky-Fuller test, and **, refers significant at 5 % levels.

**Table 3:** Vector Autoregressive (VAR) lag length selection.

Lag	Information Criteria		
	AIC	SIC	HQ
0	−9.9669	−9.8433	−9.9176
1	−19.1817	−18.5637	−18.9350
2	−19.8914*	−18.7790*	−19.4472*
3	−19.7362	−18.1294	−19.0946

Note: AIC: Akaike Information Criterion; SIC: Schwarz Information Criterion; HQ: Hann-Quinn Information Criterion.

Table 4 exposes the maximum Eigen-value test statistic, which implies the presence of one co-integrating equation that reveals a long-run relationship among the study variables. Table 5 presents the output of the normalized long-run equation. The findings show that all our predictor variables have a significant impact on total industrial production in the long run. According to the output, a 1 % increase in interest rates imposed on the large industry sector (LI) will cause a 2.817 % rise in total industrial production. That implies that, in the long run, if the imposed interest rate on large industry is increased, the large industrial output may flourish by doing good business, which will have a significant positive impact on total industrial development.

**Table 4:** Johansen co-integration test summary.

H_0_	H_A_	Eigen value	Max-Eigen Statistic	0.05 Critical Value	p-value
r = 0	r > 0	0.349935	32.30126	27.58434	0.0115**
r ≤ 1	r > 1	0.095065	7.491942	21.13162	0.9320
r ≤ 2	r > 2	0.042219	3.235191	14.26460	0.9298
r ≤ 3	r > 3	0.002146	0.161132	3.841466	0.6881

Note:** indicates significance at 5 %.

**Table 5:** Results of normalized long-run equation.

Variable	Coefficient	Standard Error	*P*- Value
LnLIWAIRA	2.817	0.4392	0.000**
LnSMEWAIRA	−2.899	0.3051	0.000**
LnINF	−1.547	0.3174	0.000**

Note: ** refers significant at 5 % levels.

Moreover, we noticed that small and medium industry has a negative and significant effect on total industrial production. Hence, the estimated long-run coefficients of −2.899 indicate that a 1 % increase in interest rates imposed on small and medium industry leads to a 2.899 % decrease in total industrial production. Besides, the inflation rate has yielded a negative and significant dominance on total industrial production. Furthermore, from following output, 1 % increases in inflation responds to 1.547 % decrease in total industrial production. A positive industrial development also refers to positive financial or economic growth [25]. From this point of view, the impact of inflation on industrial growth may line up with the result of Kim and Lin [23].

Table 6 summarizes the output of short-run dynamics. Here, the error correction term is statistically significant at 5 %. The estimated error correction term is −0.6658 and implies that short-run dynamics converge to the equilibrium point at a speed of 66.58 %. Furthermore, the findings show that only the weighted average interest rate imposed on SMEs has a negative and significant relationship with total industrial production in the short run. Therefore, it may be concluded that an immediate increase in the interest rate in SME will decrease the total industrial production by 2.04 %, which is in line with the traditional expectation [17].

**Table 6:** The Vector error correction model (VECM) test results.

Variables	Coefficients	Standard error	*P*-value
ECM_t-1_	−0.6658	0.1168459	0.000**
LnTIPI	0.0917	0.1153726	0.426
LnLIWAIRA	1.00033	0.7096661	0.159
LnSMEWAIRA	−2.03558	0.6826242	0.003**
LnINF	−0.5169591	1.44827	0.721
Constant	−0.0003317	0.0118726	0.979
R^2^	0.4067 47.98124 0.0000		
χ ^2^-statistic			
χ ^2^-statistic (probability)			

Note: ** refers significant at 5 % levels.

### 4.2 Diagnostic tests of proposed VEC model & robustness checking

**Fig. 1:**
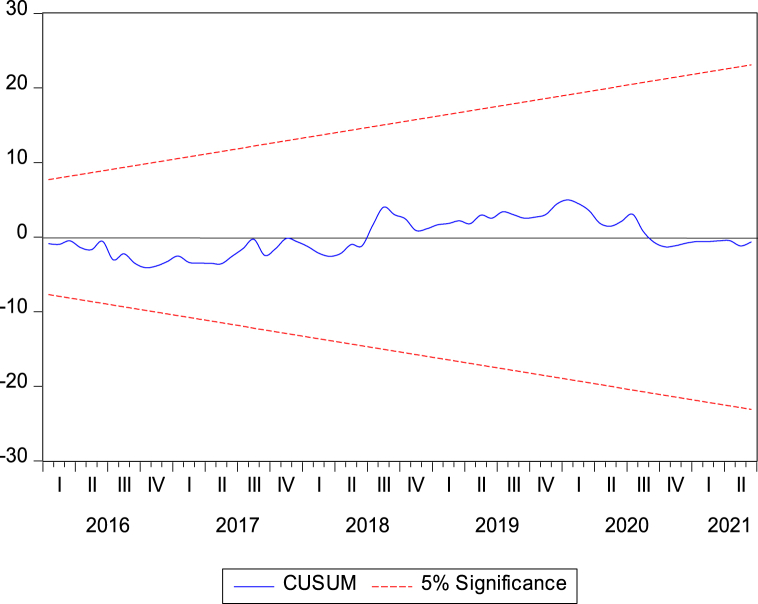
Parameter stability by CUSUM test.

**Fig. 2:**
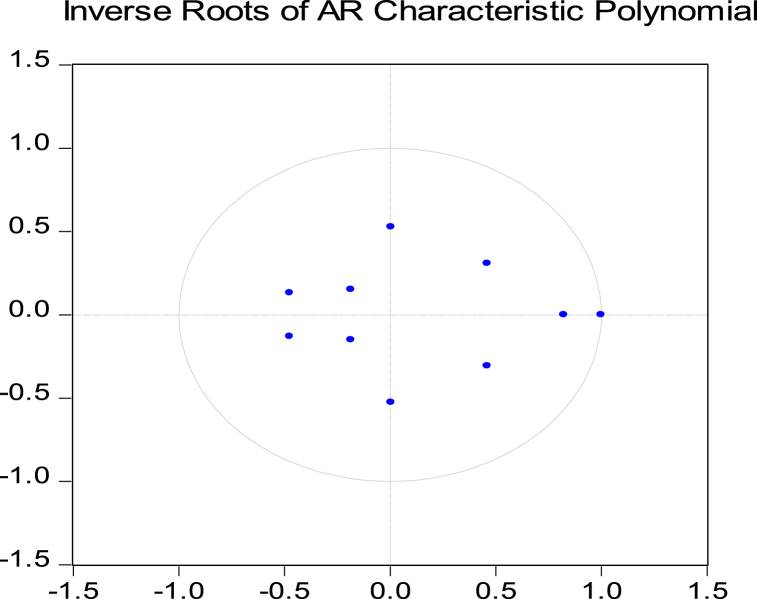
Residual stability check of VECM model.

Table 7 captures the outcomes of the diagnostic tests of our proposed VECM model. The residuals of our proposed model are free from serial correlation and normality problems. Moreover, we have applied the CUSUM test proposed by Brown et al. [42] to verify the stability of our estimated parameters, as illustrated in Fig. 1. In addition, if all roots have modulus smaller than one and remain within the unit circle, then the estimated VAR model is considered stable [43]. Fig. 2 supports this statement for our current study. Hence, we can conclude that our proposed VECM model is statistically stable.

**Table 7:** Diagnostic test results.

Test	p-value	Null Hypothesis(H_0_)	Decision
Breusch-Godfrey serial correlation LM test	0.71523	There is no autocorrelation	H_0_ accepted
Jarque-Bera(JB)	0.0000	Residuals are normally distributed	H_0_ accepted

Table 8 presents the output of the estimated VECM and FMOLS models, and the scenario of the considered macroeconomic variables toward total industrial production is almost the same.

**Table 8:** Results of long-run parameters of VECM and FMOLS method.

Variable	VECM		FMOLS	
	Coefficient [std. Error]	*P*-Value	Coefficient [std. Error]	*P*-Value
LnLIWAIRA	2.817[0.4392]	0.000**	2.624[0.4935]	0.000**
LnSMEWAIRA	−2.899[0.3051]	0.000**	−2.753[0.3447]	0.000**
LnINF	−1.547[0.3174]	0.000**	−1.444[0.3527]	0.001**

Note: ** refers significant at 5 % levels.

## 5 Conclusion

This study attempts to empirically investigate the responsiveness of industrial growth with respect to changes in interest rates on loans and the inflation rate. In addition, the evidence reveals that the interest rate on loans and the inflation rate have a considerable influence on the overall industrial growth of Bangladesh. VECM framework results in the short run imply that the interest rate imposed on SME loans has a negative and significant connection to total industrial production. A rapid increase in the interest rate on SME loans impedes the output of SMEs, which in turn results in a reduction in the SMEs' overall revenue. This is because SMEs must contend with limited production capacity while still generating revenue for their businesses and also endure some other expenses [18]. But the result becomes inconclusive for large industries in the short run.

However, the answer to the long-run equation indicates that the interest rates that are imposed on loans made to both large industries (LI) and small and medium-sized enterprises (SMEs), in addition to the inflation rate, have a considerable impact on the overall level of industrial production in Bangladesh. The conclusion that can be drawn from this is that when the rate of inflation rises, the expansion of the industrial sector will slow down as a direct consequence of the rising cost of production. In the case of the interest rate on large-scale industries, in order for the industries to be able to deal with the increasing interest rate on loans, the industries will need to, over the course of time, expand their production output [12]. An increase in the interest rate on loans given to small and medium-sized businesses (SME) has a major and detrimental effect on the expansion of industry in Bangladesh, even over the long term. An increasing rate of interest on SME loans is a burden because small and medium-sized enterprises (SME) industries already face a number of challenges, including limited production capacity, a limited workforce, limited access to better technology, and so on ([13,15]). So, low interest rates are highly recommended for developing countries like Bangladesh [44].

In light of the cited literature and empirical findings, we are able to draw the following conclusions:

1. The accessibility of credit, especially to small and medium-sized enterprises (SME) and major businesses plays an essential role in propelling industrial production and economic progress in Bangladesh.
2. Increasing the amount of credit available to small and medium-sized enterprises (SME) and major sectors at a reduced interest rate can result in increased investment, expansion, and overall output.
3. High inflation can have a detrimental effect on industrial production by increasing the cost of producing items for businesses, lowering the purchasing power of consumers, and lowering the value of loans. As a result, it is essential to maintain the rate of inflation at a general level at which investment is encouraged while also ensuring that it does not undermine the broader economy and our recommendation is the central bank should try to maintain inflation and the interest rate at a low level in the short run which in line with [45].

## 6 Recommendations

The effect that loans to small and medium-sized businesses (SME loans), loans to large industries (large industry loans), and inflation have on industrial production is complex and multifaceted. For policymakers and economists who want to promote economic growth and stability, it is important to understand the interplay between these factors. Therefore, for the policymakers of Bangladesh, it is recommended that:

1. From a macroeconomic standpoint, monetary policy should be formulated in such a way that the transition effect of the money supply and interest rates does not hamper industrial growth by increasing inflation.
2. The interest rate structure of loans should be segmented according to the purpose of the loan. That is, if credit is given to SMEs, then the interest rate should be formulated according to that firm's ability to repay the loan and still make revenue. On the other hand, for providing credit facilities to the large industrial sector, a stable interest rate structure should be maintained.

## 7 Limitations and future implications

This study is conducted using only the data of the formal banking sector in Bangladesh. But there are other organizations, such as non-bank financial institutions, co-operatives, and microcredit organizations, that provide credit facilities to SMEs and, in some cases, large industries. Due to the unavailability and incoherent structure of their data, those could not be included in this study.

Therefore, if all the data from all credit-granting organizations could be included, it would give a much better picture of the overall situation in Bangladesh and help policymakers formulate more effective policies. Again, there is scope to calculate the optimum level of inflation rate for maximum industrial production in the context of Bangladesh.

## Data availability

1. The data of WAIR is available in Bangladesh Bank's NSDP portal in Interest Rates tab in SDMX format and also in IMF's database. Statistics Department of Bangladesh Bank is the sole owner of the data.

https://www.bb.org.bd/econdata/nsdp/nsdp_bb.php.

The data can also be made available on request.

2. The data of inflation rate and total industrial production index is available on “Monthly economic trends” published by Bangladesh Bank.

https://www.bb.org.bd/en/index.php/publication/publictn/3/10.

## Funding

The authors declare that no funds, grants, or other support were received during the preparation of this manuscript.

## Research data policy

The data is collected by Statistics Department of Bangladesh Bank according to “Spread Calculation Procedure” available in the guidelines section on the website of Bangladesh Bank.

https://www.bb.org.bd/en/index.php/about/guidelist.

## CRediT authorship contribution statement

**Adnan Chowdhury:** Conceptualization, Data curation, Formal analysis, Investigation. **Tamanna Siddiqua Ratna:** Data curation, Formal analysis, Investigation, Software, Writing – original draft, Writing – review & editing. **Tanzin Akhter:** Investigation, Methodology, Writing – original draft. **Syed Far Abid Hossain:** Conceptualization, Supervision, Writing – review & editing.

## Declaration of competing interest

The authors declare that they have no known competing financial interests or personal relationships that could have appeared to influence the work reported in this paper.
